# Artificial Intelligence-Based Evaluation of Post-Procedural Electrocardiographic Parameters to Identify Patients at Risk of Atrial Fibrillation Recurrence After Transcatheter Ablation

**DOI:** 10.3390/jcm14228248

**Published:** 2025-11-20

**Authors:** Gennaro De Rosa, Marco Giuggia, Mattia Peyracchia, Martina Peddis, Roberto Di Summa, Elisa Pelissero, Giuseppe Trapani, Davide De Los Rios, Fabio Ugliano, Plinio Cirillo, Gaetano Senatore

**Affiliations:** 1 Department of Advanced Biomedical Sciences, Division of Cardiology, University of Naples Federico II, 80131 Naples, Italy; gennaro.derosa3@unina.it; 2 Ciriè Hospital, 10073 Ciriè, Italy; mgiuggia@aslto4.piemonte.it (M.G.); mpeyracchia@aslto4.piemonte.it (M.P.); mpeddis@aslto4.piemonte.it (M.P.); rdisumma@aslto4.piemonte.it (R.D.S.); epelissero@aslto4.piemonte.it (E.P.); gtrapani@aslto4.piemonte.it (G.T.); gsenatore@aslto4.piemonte.it (G.S.); 3 Department of Medicine, University of Naples Federico II, 80131 Naples, Italy; d.delosrios@studenti.unina.it (D.D.L.R.); fa.ugliano@studenti.unina.it (F.U.)

**Keywords:** atrial fibrillation, catheter ablation, artificial intelligence, electrocardiography, P-wave, risk stratification, recurrence prediction

## Abstract

**Background/Objectives:** Arrhythmic recurrence is a common issue affecting a significant percentage of patients undergoing transcatheter ablation (TCA) of Atrial Fibrillation (AF). The use of artificial intelligence (AI) for the identification of electrocardiographic predictors of post-ablation recurrence may offer a valuable and cost-effective approach to improve risk stratification and optimize follow-up. This study aims to investigate the relationship between post-procedural electrocardiographic (ECG) P-wave parameters, measured using AI, and AF recurrence in patients undergoing transcatheter ablation (TCA). **Methods:** Seventy-four patients (age 62.36 ± 10.4 years) with a diagnosis of AF were retrospectively analyzed. ECGs were processed using AI software to analyze P-wave-related variables. All patients had either an implantable loop recorder (ILR) or another form of cardiac implantable electronic device (CIED). **Results:** Post-procedural P-wave amplitude in lead II (PwA in lead II) showed a significant association with AF recurrence, defined as an average arrhythmic burden >6% at one-year follow-up. **Conclusions:** These findings underscore the potential of PwA in lead II as a biomarker for the follow-up of patients undergoing TCA and highlight the contribution of AI in the analysis of electrocardiographic parameters predictive of AF recurrence. Together, these results may contribute to the development of early risk-stratification strategies following catheter ablation.

## 1. Introduction

Arrhythmic recurrence is a well-recognized limitation of transcatheter ablation (TCA) for atrial fibrillation (AF), affecting a substantial proportion of treated patients, with estimated rates ranging from 20% to 50% within the first year [1]. Identifying predictors of post-ablation recurrence is crucial for improving risk stratification and optimizing follow-up strategies. Several factors have been examined to identify patients at increased risk of recurrence and to support a more individualized therapeutic approach. 

Previous studies [2] have indicated that different electrocardiographic parameters may be associated with the risk of arrhythmic recurrence, including P-wave duration and amplitude. Artificial intelligence (AI) represents an innovative tool for improving risk stratification and predicting AF recurrence after transcatheter ablation, owing to its ability to analyze large volumes of complex data. AI can automate ECG measurements, detect subtle or multidimensional patterns and enhance human interpretation. 

AI-based software has already been applied to large electrocardiographic datasets [3,4,5,6,7] for AF detection and prediction; however, evidence supporting its use in patients who have already undergone TCA remains limited. Therefore, the aim of this study was to identify potential post-procedural electrocardiographic predictors of arrhythmic recurrence one year after the index procedure using AI-assisted analysis.

## 2. Materials and Methods

### 2.1. Study Population and Procedural Workflow

A total of 74 patients met the inclusion criteria and underwent TCA for AF in 2023 at the Complex Operative Unit (COU) of Ciriè Hospital, ASL TO4 (Turin); 58 (78.4%) were men and 16 (21.6%) were women. Baseline characteristics of the enrolled patients are reported in Table 1.

Patients were eligible for inclusion if they met all the following criteria: age ≥18 years; documented diagnosis of atrial fibrillation (paroxysmal, persistent or long-standing persistent); undergoing catheter ablation for AF at the participating center during the study period; availability of a standard 12-lead ECG in sinus rhythm, recorded within the first hours following the ablation procedure; presence of an implantable loop recorder (ILR) or other cardiac implantable electronic device (CIED) with continuous rhythm-monitoring capability during follow-up.

Patients were excluded if they met any of the following criteria: absence of a post-procedural ECG in sinus rhythm suitable for analysis; lack of an ILR or CIED capable of storing and transmitting AF burden data; previous atrial arrhythmia surgery or non–pulmonary vein ablation procedures altering atrial anatomy; poor-quality ECG tracings preventing reliable P-wave assessment; incomplete clinical or follow-up data.

Radiofrequency (RF) was the predominant ablation modality, accounting for 53 procedures (73%), whereas cryoenergy and laser energy were used in 14 (18.9%) and 7 (9.5%) cases, respectively. The mean age was 62.36 ± 10.41 years (range 32–83).

At hospital admission, 36 (48.6%) patients had paroxysmal atrial fibrillation, while 38 patients (51.4%) presented with persistent atrial fibrillation. A total of 55 (74.3%) patients were treatment-naïve for transcatheter ablation (TCA), while 12 (16.2%) patients underwent simultaneous cavotricuspid isthmus (CTI) ablation.

Most patients (69; 93.2%) had an implantable loop recorder (ILR) while 5 patients (6.8%) had another type of cardiac implantable electronic device (CIED). All patients were admitted to the hospital at least one day before the scheduled procedure and underwent routine laboratory testing, standard 12-lead ECG, chest X-ray, transesophageal echocardiography (TEE) and a pre-procedural anesthesiology assessment. The standard protocol for TCA involved general anesthesia and dual femoral venous access with placement of a steerable catheter in the coronary sinus (CS). Subsequently a fluoroscopy-guided transseptal puncture was performed and pulmonary vein isolation (PVI) was achieved.

Immediately after the procedure, all patients underwent transthoracic echocardiography (TTE) to exclude pericardial effusion. A follow-up visit was scheduled within the following 3 months. All patients were provided with a dedicated application enabling remote monitoring of CIED. All device models included an automatic transmission function for arrhythmic recurrence alerts. Expert nurses performed weekly analyses of alarm notifications, in collaboration with the reference electrophysiologist, and generated a comprehensive report every three months.

### 2.2. AI Analysis

#### 2.2.1. Image Preparation and Calibration

The processing pipeline followed a structured workflow consisting of:

- ECG digitization;
- image upscaling;
- grid-based pixel-to-unit calibration;
- deterministic AI-based waveform measurement;
- quality-control validation against manual measurements.

ECG images were first enhanced using an online artificial intelligence tool (Pixelcut, 490 43rd Street Suite 210, Oakland, CA 94609, USA) with a four-fold resolution enhancement to improve grid visibility. To ensure that the upscaling process did not introduce interpolation artifacts or altered waveform morphology, a random subset of tracings was manually remeasured before and after enhancement (P-wave amplitude and duration, PR interval, QRS duration, QT interval). Deviations were consistently <1%, confirming preservation of waveform integrity. No de-noising, smoothing or waveform-modifying filters were applied at any stage.

#### 2.2.2. AI ECG Measurement Pipeline

The optimized ECG images were analyzed using a deterministic large-language-model workflow (GPT-5, OpenAI; temperature = 0, top_p = 1), designed to produce fully reproducible and rule-based measurements. The model did not infer ECG features autonomously but executed predefined analytic instructions embedded within a structured prompt (full system and task prompts provided in Supplementary Methods S1).

Preprocessing and calibration

Pixel-to-millimeter calibration was derived from the 1 × 1 mm ECG grid. Horizontal and vertical pixel densities were computed independently, enabling conversion to:

- time (ms) at 25 mm/s;
- amplitude (mV) at 10 mm/mV.

Rule-Based Wave Identification

Wave boundaries followed explicit electrophysiologic definitions:

- P-wave onset: first deviation from the isoelectric line preceding the QRS;
- P-wave offset: return to the isoelectric line before the PQ junction;
- QRS complex: maximal-slope depolarization, used as temporal reference;
- T wave: post-QRS repolarization, excluded via a pre-QRS search window.

The algorithm executed the following steps in each lead:

1. QRS complex detection using slope- and energy-based criteria;
2. Opening of a 250 ms backward search window to locate the P wave;
3. Baseline estimation via the median of a 120–160 ms low-slope segment;
4. Identification of P-wave onset and offset based on baseline crossings.

Measurement Protocol

For each lead, the model measured:

- P-wave duration: onset-to-offset interval (ms);
- P-wave amplitude: maximal absolute deviation from the baseline (mV).

Five consecutive sinus beats were analyzed in each lead, and the arithmetic mean was used as the final measurement. Values were reported with clinically appropriate resolution (0.01 mV and 4 ms).

Derived Indices

From the 12-lead dataset, the following indices were computed:

- mean P-wave amplitude;
- maximum P-wave amplitude;
- P-wave dispersion (max–min duration across limb, precordial and all leads);
- P-wave Vector Magnitude (PwVM):

$$\sqrt{PwA_{DII}^{2}+PwA_{V6}^{2}+(0.5\times PwA_{V2}{)}^{2}}$$

Leads in which the P wave could not be reliably identified were flagged and excluded from the corresponding metric.

Reproducibility and Documentation

All analytical components—including calibration parameters, conversion formulas, system/task prompts, rule definitions, JSON output schema and an example model output—are fully documented in Supplementary Methods S1, ensuring complete reproducibility by external investigators.

#### 2.2.3. Quality Control and Validation 

AI-generated measurements were compared with manual measurements obtained using EP-Calipers. The analysis was rerun on a random subset to confirm complete intra-observer reproducibility, which was ensured by the model’s deterministic configuration. Inter-observer reliability was evaluated by comparing AI-derived and manual values; discrepancies > 8 ms (for duration) or >0.02 mV (for amplitude) triggered manual review and verification.

### 2.3. Classification of Arrhythmic Recurrence 

We reviewed and downloaded the device-recorded data on atrial fibrillation (AF) episodes during the 12-month follow-up after the index ablation. Based on these recordings, each patient was assigned to one of three categories. Patients with no documented episodes of sustained AF during the entire follow-up were classified as having no arrhythmic recurrence. Those who experienced AF episodes but maintained an AF burden below 6% were categorized as having non-significant burden recurrence. Finally, patients whose device diagnostics reported an AF burden exceeding 6% were classified as having significant burden recurrence. This classification was derived exclusively from continuous monitoring through ILRs or CIEDs. To avoid defining arrhythmic-recurrence severity arbitrarily, we performed an exploratory ROC analysis (Figure S1) using AF burden as the outcome and P-wave amplitude in lead II as the predictor. The ROC curve demonstrated good discriminatory ability (AUC = 0.772) and the sensitivity–specificity trend showed a clear inflection point around the 6% burden level. This indicates that values exceeding 6% identify a group with a distinct arrhythmic profile compared with patients below this threshold.

Based on this empirical behavior of our dataset, the >6% cutoff was adopted as the operational definition of clinically relevant recurrence for subsequent analyses.

### 2.4. Statistical Analysis 

For the analysis of normally distributed variables, the mean ± standard deviation was used as a measure of central tendency and dispersion. For variables not normally distributed, the median (range min-max) was used. For binary and ordinal variables, absolute and relative frequencies (percentages) were expressed. 

Correlation analyses were performed using Spearman’s Rho (ρ) tests, as well as multivariate logistic regression models. In the logistic regression model, predictors of clinically relevant AF recurrence (AF burden > 6%) were expressed as Odds Ratios (ORs) with 95% confidence intervals (CIs), using clinically meaningful increments for continuous variables (e.g., 0.1 mV for P-wave amplitude). Multicollinearity was assessed through the Variance Inflation Factor (VIF), with values < 2 indicating the absence of significant collinearity. Model calibration and fit were evaluated using the Hosmer–Lemeshow goodness-of-fit test, with statistical significance set at *p* < 0.05. 

To account for the limited sample size (*n* = 74) and avoid overfitting, the multivariate logistic regression model was deliberately restricted to a small number of clinically relevant predictors, in accordance with accepted events-per-variable (EPV) criteria. A post hoc power assessment confirmed that the observed effect size for P-wave amplitude in lead II provided adequate statistical power within this dataset. In addition, a sensitivity analysis was performed using penalized logistic regression (LASSO), which consistently identified P-wave amplitude in lead II as the strongest predictor of arrhythmic recurrence, supporting the robustness of the findings. The results were obtained using IBM SPSS Statistics (Version 28, IBM Corp., Armonk, NY, USA).

### 2.5. Data Privacy and Ethical Handling of ECG Images

All ECGs were fully de-identified prior to AI processing. Patient identifiers, exam dates, device serial numbers, and any metadata linking the tracing to an individual subject were removed before image export. The upscaling step (Pixelcut) and the AI-based measurement workflow (ChatGPT) were performed exclusively on de-identified images. No clinical or demographic information was transferred.

Pixelcut processed the images via its cloud-based server using de-identified graphical files only, with no possibility of patient re-identification. ChatGPT analysis was performed exclusively on de-identified ECG images. According to the platform’s privacy safeguards, files are processed only transiently during the session and are neither stored nor used to train models. No clinical or personal data were entered, ensuring full compliance with GDPR and institutional privacy requirements.

All procedures adhered to institutional privacy policies, GDPR regulations and local ethical standards governing the use of third-party software for research purposes. 

## 3. Results

Bivariate analyses identified several post-procedural ECG parameters that were significantly associated with atrial fibrillation (AF) recurrence. The complete set of correlation coefficients is presented in Table 2.

**Figure 1:**
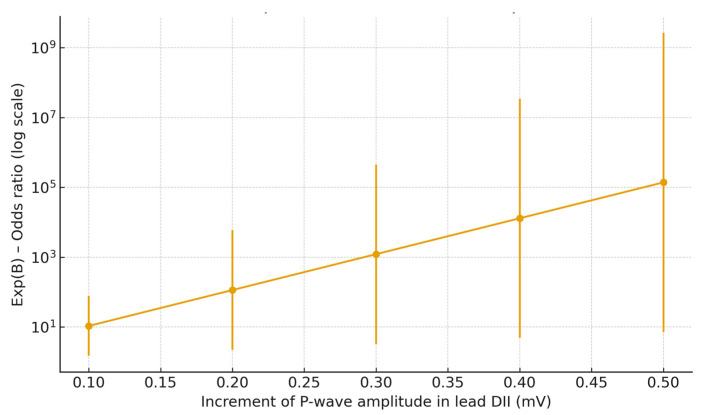
Incremental effect of P-wave amplitude in lead DII on AF recurrence.

Significant correlations with AF recurrence were observed for P-wave duration in lead V6, P-wave vector magnitude (PwVM), P-wave amplitude in leads II and III and mean P-wave amplitude. When patients were stratified by AF burden > 6%, additional associations emerged for P-wave duration in lead III, P-wave duration in lead V6, P-wave amplitude in lead II and mean P-wave amplitude. In the multivariate logistic regression model, P-wave amplitude in lead II remained the only independent predictor of clinically relevant AF recurrence with AF burden > 6% (odds ratio [OR] and confidence intervals [CI] are reported in Figure 1 and Supplementary Table S1).

Odds ratios (Exp(B)) are shown for 0.1 mV increments in P-wave amplitude; error bars represent 95% confidence intervals. A logarithmic scale was applied to the y-axis for visualization. Higher P-wave amplitude was associated with increased odds of AF recurrence (OR per 0.1 mV = 1.27, 95% CI 1.04–1.58, *p* = 0.019).

**Figure 2:**
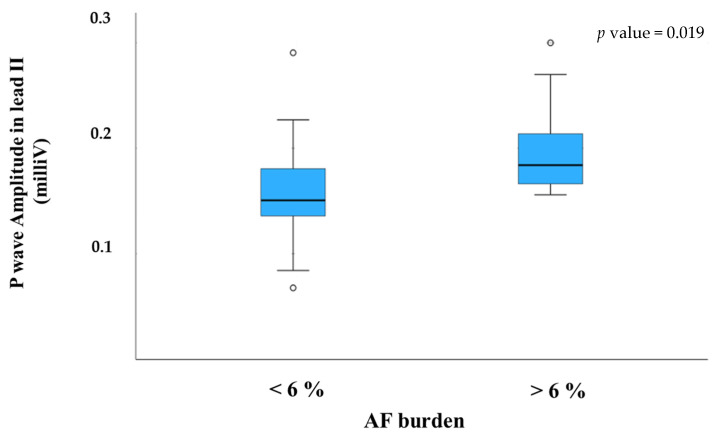
Boxplot illustrating P-wave amplitude in lead II stratified by AF burden.

A boxplot illustrating the distribution of P-wave amplitude in lead II according to AF burden is shown in Figure 2. A complementary scatter plot depicting the same relationship is provided in the Supplementary Materials (Figure S2).

Boxplot demonstrating the significant difference in P-wave amplitude between patients with AF burden ≤6% and those with AF burden >6% at one-year follow-up (*p* < 0.01). Outliers were excluded for clarity.

## 4. Discussion

Catheter ablation is a well-established therapy for atrial fibrillation (AF), especially in symptomatic patients refractory to drug therapy [8,9,10,11]. However, a significant percentage of patients experience arrhythmic recurrence after the procedure, requiring further interventions or treatment modifications. Recurrence rates vary depending on patient characteristics, AF type and ablation techniques used. 

Haïssaguerre et al. [12] demonstrated that pulmonary vein isolation (PVI) is the cornerstone of catheter ablation for AF, yet a substantial proportion of patients still experience recurrence. Similarly, Verma et al. [13] reported that up to 40% of patients with persistent AF have recurrences following initial ablation. 

P-wave duration, amplitude and dispersion have been identified as predictors of arrhythmic recurrence. In particular, a meta-analysis by Intzes et al. [2] found that P-wave duration was a strong predictor of recurrence following transcatheter ablation for AF. Another meta-analysis by Liu et al. [14] showed that P-wave dispersion is a useful marker for predicting AF recurrence, with higher values correlating with poorer outcomes post-ablation. Additionally, Park et al. [15] identified P-wave amplitude in lead II as an important predictor of recurrence, with values below 0.1 mV significantly associated with higher recurrence rates. 

In this context, our work—although limited in terms of sample size—may be considered pioneering, as it aims to leverage the vast potential of AI software for ultra-sensitive analysis of electrocardiographic parameters predictive of arrhythmic recurrence in the immediate post-ablation phase.

Recent studies employing deep-learning-based ECG algorithms have demonstrated promising performance in predicting AF recurrence and in identifying atrial remodeling patterns beyond visual inspection. Models integrating convolutional neural networks, multimodal ECG–CMR frameworks or time–frequency representation learning have shown good discriminatory ability and have highlighted the potential of automated feature extraction. For instance, multimodal deep-learning frameworks [16] that combine structured ECG features with imaging biomarkers have achieved superior predictive performance compared with traditional clinical or ECG-only models.

Our analytical approach differs substantially from these deep learning systems: instead of latent feature extraction, it relies on deterministic rule-based morphological measurements. This makes the analysis fully transparent, reproducible and clinically interpretable, although potentially less powerful than fully data-driven models. 

Our study provides a form of early risk stratification that differs from existing deep-learning–based ECG models. Recent AI–ECG studies have primarily focused on pre-procedural or late post-procedural tracings, often relying on complex latent-feature extraction that limits interpretability. In contrast, our approach analyzes ECGs recorded within minutes after pulmonary vein isolation, at a time point largely unexplored in prior literature. 

This immediate post-ablation window may capture acute electrophysiological disturbances that anticipate later remodeling processes. Moreover, our deterministic, rule-based workflow provides full transparency and reproducibility, facilitating clinical verification and lowering the barrier for real-world implementation. These aspects differentiate our method from more opaque deep-learning frameworks and highlight its potential as an accessible and clinically interpretable early biomarker-extraction tool.

Interpretability remains a key challenge in AI-driven electrophysiology. Techniques such as saliency maps, gradient-based visualization, and SHAP (Shapley Additive Explanations) have recently been introduced to improve transparency by highlighting ECG regions that contribute most to model decisions. These methods enhance clinical explainability and facilitate validation of AI-derived insights. Future studies could incorporate similar approaches to complement the high-resolution waveform analysis used in our investigation.

Finally, recent work [17] has demonstrated the growing impact of AI in electrophysiology and arrhythmia management. Successful applications of AI in identifying arrhythmic substrates and guiding EP workflows have been reported, reinforcing the progressive integration of advanced computational tools into clinical practice. Incorporating these developments helps contextualize our findings within the broader landscape of emerging AI techniques in cardiac electrophysiology.

It is increasingly evident that there is a need to identify clinico-instrumental markers capable of strongly predicting clinically relevant arrhythmic recurrence in patients undergoing catheter ablation. In this context, ECG parameters capable of predicting recurrence would be invaluable, given the ease of access to the method and minimal costs.

The patient population included individuals treated with different energy modalities, allowing us to demonstrate that the ECG parameters examined—particularly P-wave indices—are independent of the ablation technique employed. Both bivariate and multivariate analyses consistently identified P-wave amplitude in lead II as the strongest predictor of AF recurrence at one year, reinforcing its value as a clinically meaningful marker.

Our findings are consistent with previous reports; Park et al. [15] have shown that P-wave amplitude in lead II is a reliable marker of post-ablation recurrence, with lower amplitudes associated with worse outcomes. Physiologically, the normal range for P-wave amplitude in lead II lies between 0.1 and 0.25 mV [18]. Values below 0.1 mV may reflect atrial fibrosis, reduced atrial muscle mass or impaired conduction, all of which predispose to recurrence after trans-catheter ablation [15,19]. Low-amplitude P waves are often accompanied by prolonged P-wave duration (>120 ms), a combination that has been traditionally associated with pathological atrial remodeling.

Conversely, an increase in P-wave amplitude immediately after ablation has been interpreted as a sign of improved atrial conduction and more homogeneous depolarization, correlating with a lower recurrence risk [20,21,22]. However, very high P-wave amplitudes may instead reflect heterogeneous conduction or early micro-reentrant activity within partially remodeled atrial tissue [23,24,25]. These observations align with our results, in which higher P-wave amplitude in lead II was positively correlated with clinically significant AF recurrence (AF burden > 6%) in both bivariate and multivariate analyses.

A plausible electrophysiological interpretation for this finding is that areas of incomplete lesion formation or heterogeneous conduction—commonly observed in the early post-ablation phase—may alter atrial activation patterns in a way that increases the net depolarization energy recorded on the surface ECG. In this context, higher P-wave amplitude in lead II could reflect residual zones of slowed conduction or micro-reentrant activity occurring at the interface between healthy and partially remodeled tissue. These phenomena have been described in studies using high-density mapping and imaging modalities [26], where transition zones between fibrosis and viable myocardium may generate fragmented or amplified local activation patterns. 

Conversely, when fibrosis becomes more advanced and conduction markedly disorganized, P-wave amplitude may instead decrease, sometimes accompanied by visible fragmentation, as reported in voltage-mapping and late-enhancement MRI studies [27,28].

Because our analysis was limited to surface ECGs, these mechanistic interpretations should be considered hypothesis-generating and further correlation with high-resolution mapping or structural imaging is warranted. 

Importantly, the surface ECGs in our study were recorded immediately after ablation, before structural remodeling or reverse remodeling could occur. At this stage, acute electrophysiological disturbances—rather than chronic architectural changes—likely predominate. This temporal context may explain why P-wave amplitude in lead II emerged as a stronger predictor than parameters such as P-wave dispersion, which may require more advanced or chronic remodeling to become evident. Our findings therefore suggest that post-procedural P-wave amplitude may serve as an early indicator of an atrial substrate prone to later modification and arrhythmic recurrence.

## 5. Limitations

This study has several limitations that should be acknowledged. First, the analysis was conducted in a single center and included a relatively small sample size, which may limit the generalizability of the findings. Although all patients underwent systematic and continuous monitoring through ILRs or CIEDs, the cohort size reduces the power to detect weaker associations and increases the risk of overfitting in multivariate models.

In addition, because only patients with ILRs or CIEDs were eligible, a degree of selection bias cannot be excluded, as individuals without continuous monitoring were not represented. Although a post hoc power assessment indicated adequate power for detecting the observed effect of P-wave amplitude, the sample size remains insufficient for identifying smaller associations or interaction effects. These factors limit the external validity of the findings, underscoring the need for confirmation in larger, prospective, multicenter cohorts with broader clinical representation.

Second, ECG measurements were performed on scanned or image-exported 12-lead ECGs rather than directly on digital ECG signal files. Although we applied a rigorous pixel-to-millimeter calibration process and implemented a hybrid human–AI measurement workflow with systematic quality control, small inaccuracies inherent to image-based analysis cannot be entirely excluded.

Third, although our results suggest that P-wave amplitude in lead II is a strong predictor of AF recurrence, this parameter may be influenced by factors not assessed in the present study, such as atrial size, localized fibrosis distribution or concomitant conduction abnormalities. Advanced imaging or high-density mapping data were not available to correlate ECG findings with structural remodeling.

Finally, while the AI-assisted workflow ensured standardization and reproducibility, the algorithm relied on rule-based definitions implemented through large-language-model prompting rather than direct signal-level machine learning. More sophisticated ECG-processing frameworks that integrate signal-based deep-learning models may provide additional accuracy and should be explored in future studies.

## 6. Conclusions

In conclusion, we have shown that the P-wave amplitude in lead II, measured in the immediate post-procedural phase, may serve as a valuable biomarker associated with atrial fibrillation (AF) recurrence following transcatheter ablation. These results lay the groundwork for further prospective studies to confirm the association between P-wave amplitude and arrhythmic recurrence, and to explore the potential of targeted interventions aimed at reducing recurrence risk. 

The identification of reliable ECG biomarkers, particularly those assessable in the early post-procedural period, may facilitate personalized follow-up strategies and ultimately improve patient outcomes while reducing the need for repeat interventions. Additionally, the integration of AI-based analysis may enhance risk stratification by processing large ECG and imaging datasets, thereby improving the accuracy of recurrence assessment. 

## Figures and Tables

**Table 1:** Baseline features of the study population.

Category	Frequency	Percent
Paroxysmal AF	36	48.6
Persistent AF	38	51.4
Male	58	78.4
Female	16	21.6
Family history of CAD	10	13.5
Dyslipidemia	31	41.9
Current smoker	11	14.9
Hypertension	46	62.2
Diabetes	12	16.2
Diabetes on Insulin	1	1.4
COPD	5	6.8
Sleep apnea	1	1.4
PAD	2	2.7
Previous Stroke or TIA	6	8.1
Previous MI	5	6.8
CKD	6	8.1
Heart failure	10	13.5
Previous PCI	6	8.1
Previous CABG	2	2.7
Dysthyroidism	9	12.2

Abbreviations: AF, atrial fibrillation; CABG, coronary artery bypass graft; CAD, coronary artery disease; CKD, chronic kidney disease; COPD, chronic obstructive pulmonary disease; MI, myocardial infarction; PAD, peripheral artery disease; PCI, percutaneous coronary intervention; TIA, transient ischemic attack.

**Table 2:** Bivariate analysis of variables associated with AF recurrence (Burden > 6%).

Variable	Correlation Coefficient (ρ)	*p* Value
P-wave duration in V6	0.391	0.001
PVM	0.228	0.036
P-wave amplitude in lead II	0.389	0.002
P-wave amplitude in lead III	0.256	0.027
Mean PWA	0.308	0.010
P-wave duration in lead I	0.043	0.376
P-wave duration in lead III	0.126	0.175
P-wave duration in lead aVR	0.207	0.061
P-wave duration in lead aVL	0.220	0.050
P-wave amplitude in lead I	0.130	0.167
P-wave amplitude in lead aVL	0.160	0.117
P-wave amplitude in lead V1	0.188	0.081
P-wave amplitude in lead V6	0.150	0.134
QT dispersion	0.281	0.053

Abbreviations: PVM, P-wave vector magnitude; Mean PWA, Mean P-wave amplitude.

## Data Availability

The data that support the findings of this study are available from the corresponding author upon reasonable request. The data are not publicly available due to patient privacy and institutional ethical restrictions.

## Supplementary Materials

The following supporting information can be downloaded at: https://www.mdpi.com/article/10.3390/jcm14228248/s1, Figure S1: ROC curve of P-wave amplitude in lead II for predicting high-burden AF recurrence (>6%); Figure S2: Correlation scatter plot between P-wave amplitude in lead II and arrhythmic recurrence with AF burden >6%; Table S1: Incremental effect of P-wave amplitude in lead DII on AF recurrence; Supplementary Methods S1: AI-Based ECG Analysis Workflow.

## Abbreviations

The following abbreviations are used in this manuscript:

AF	Atrial Fibrillation
AI	Artificial Intelligence
CA	Catheter Ablation
CIED	Cardiac Implantable Electronic Device
CMR	Cardiac Magnetic Resonance
COU	Complex Operative Unit
CS	Coronary sinus
DOAC	Direct Oral Anticoagulant
ECG	Electrocardiogram
ILR	Implantable Loop Recorder
LA	Left Atrium
MI	Mitral Isthmus
MRI	Magnetic Resonance Imaging
PN	Phrenic Nerve
PVI	Pulmonary Vein Isolation
PwA	P-wave Amplitude
PwVM	P-wave Vector Magnitude
RF	Radiofrequency
SR	Sinus Rhythm
TCA	Transcatheter Ablation
TTE	Transthoracic Echocardiography
