# Supplementary material for: Artificial Intelligence-Based Evaluation of Post-Procedural Electrocardiographic Parameters to Identify Patients at Risk of Atrial Fibrillation Recurrence After Transcatheter Ablation
Source: J Clin Med. 2025 Nov 20;14(22):8248. doi: 10.3390/jcm14228248 (PMC12653835; doi:10.3390/jcm14228248)
Supplement: Supplementary file 1 [file jcm-14-08248-s001.zip › jcm-3962775-supplementary/Figure S1.pdf]

Supplementary Figure S1. ROC curve of P-wave amplitude in lead II for predicting high-burden AF recurrence (>6%)

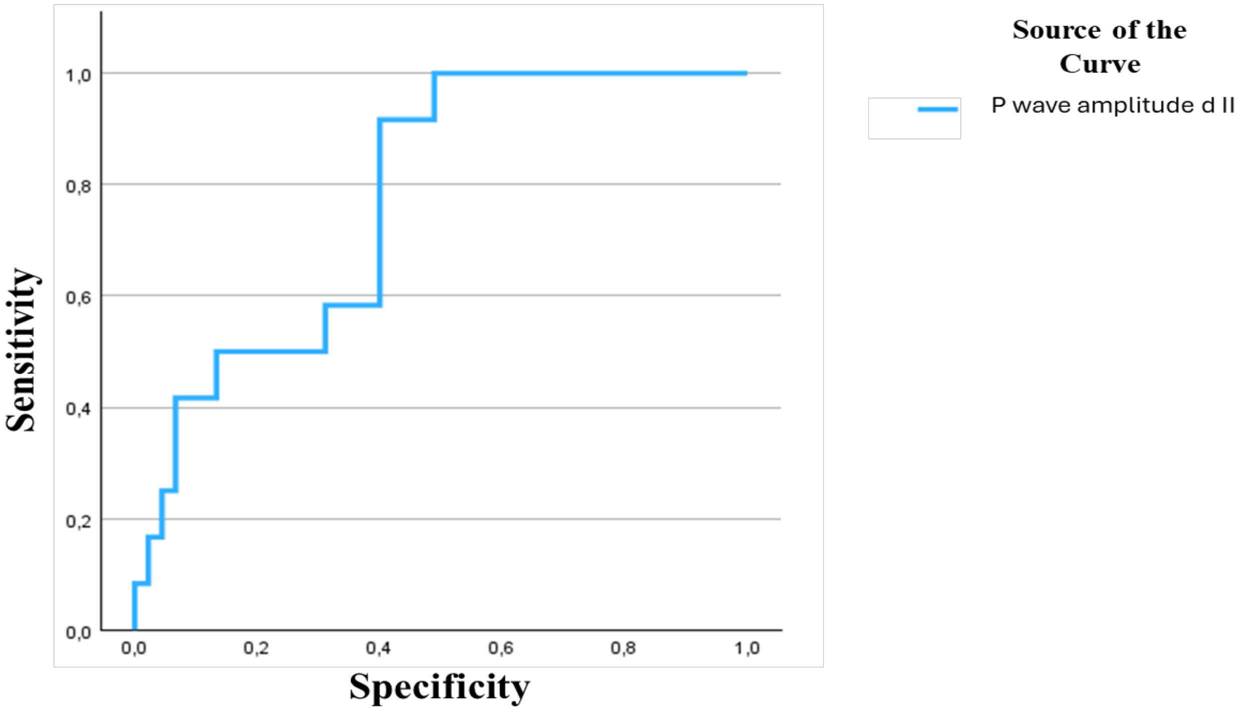

The ROC curve illustrates the discriminative ability of post-procedural P-wave amplitude in lead II to identify patients with clinically significant atrial fibrillation recurrence, defined as a daily AF burden greater than 6%.

The area under the curve (AUC) is 0.772, indicating good predictive performance.

Axes: Sensitivity (Y-axis) and 1 – Specificity (X-axis).

Legend: P-wave amplitude (Lead II).

Abbreviations: AF, atrial fibrillation; AUC, area under the curve.
