# Supplementary material for: Artificial Intelligence-Based Evaluation of Post-Procedural Electrocardiographic Parameters to Identify Patients at Risk of Atrial Fibrillation Recurrence After Transcatheter Ablation
Source: J Clin Med. 2025 Nov 20;14(22):8248. doi: 10.3390/jcm14228248 (PMC12653835; doi:10.3390/jcm14228248)
Supplement: Supplementary file 1 [file jcm-14-08248-s001.zip › jcm-3962775-supplementary/Figure S2.pdf]

Figure S2. Correlation scatter plot between P-wave amplitude in lead II and arrhythmic recurrence with AF burden >6%

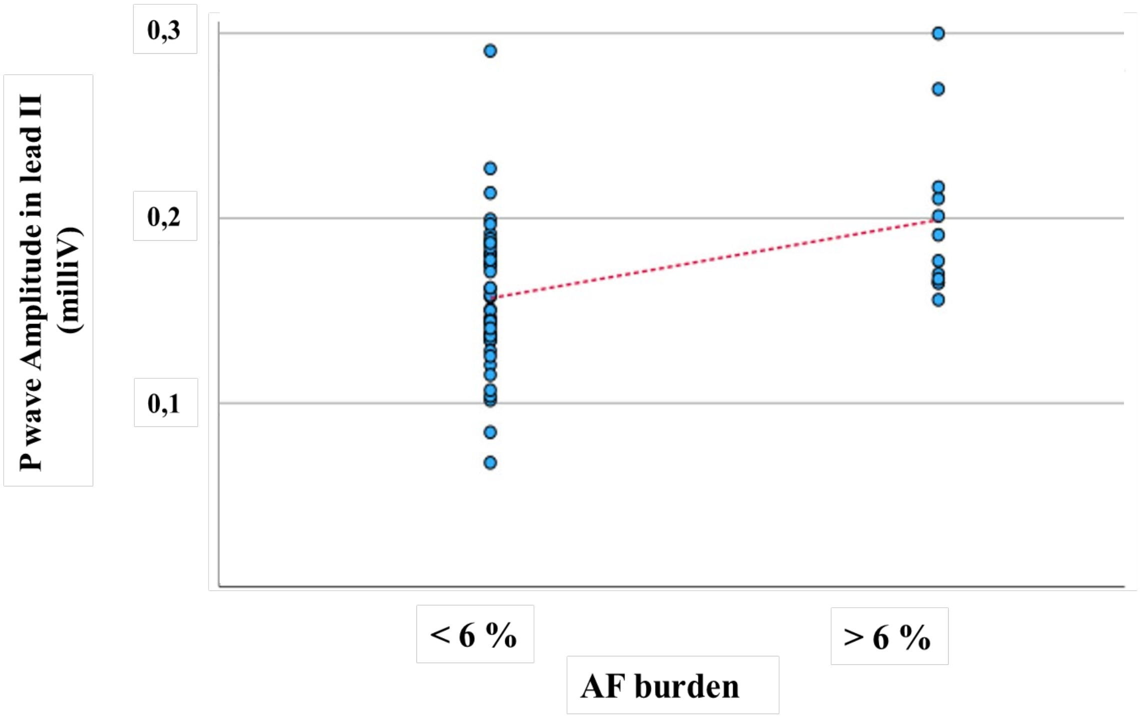

Scatter plot with trend line illustrating the number of observations per case; once again, a statistically significant correlation ($p < 0.01$) is evident between P-wave amplitude in lead II and arrhythmic recurrence with an AF burden >6%.
