# Supplementary material for: Artificial Intelligence-Based Evaluation of Post-Procedural Electrocardiographic Parameters to Identify Patients at Risk of Atrial Fibrillation Recurrence After Transcatheter Ablation
Source: J Clin Med. 2025 Nov 20;14(22):8248. doi: 10.3390/jcm14228248 (PMC12653835; doi:10.3390/jcm14228248)
Supplement: Supplementary file 1 [file jcm-14-08248-s001.zip › jcm-3962775-supplementary/Supplementary Methods S1.pdf]

Supplementary Material S1. AI-Based ECG Analysis Workflow

S1.1. Overview

A standardized artificial intelligence (AI) pipeline was used to perform quantitative measurements of the P-wave across all 12 ECG leads. The workflow consisted of the following steps:

(1) ECG image digitalization;
(2) image upscaling;
(3) grid-based pixel-to-unit calibration;
(4) deterministic AI-based waveform measurement;
(5) quality-control validation against manual EP-Calipers measurements.

All procedures were executed using a fully reproducible prompting system (GPT-5, deterministic mode), with temperature = 0 and top_p = 1.

S1.2. AI System Configuration

The ECGs were measured using the OpenAI large language model (LLM; GPT-5), operating in deterministic analytical mode. Stochastic sampling was disabled (temperature = 0, top_p = 1) to ensure reproducibility. The model received a fixed system prompt defining measurement rules and a structured task prompt providing calibration values and file descriptors.

No filtering, smoothing, or signal-altering transformations were applied at any stage.

S1.3. ECG Upscaling

Images were upscaled four-fold using Pixelcut (Pixelcut, Oakland, CA, USA). The software applies a resolution-enhancement algorithm without modifying waveform morphology. Validation of this step included manual pre- and post-processing comparisons of P-wave amplitude, P-wave duration, PR interval, QRS duration, and QT interval, demonstrating <1% deviation.

S1.4. Pixel-to-Unit Calibration

Calibration was performed using the intrinsic ECG grid.
Paper speed: 25 mm/s.
Voltage gain: 10 mm/mV.
Small square: 1 mm × 1 mm.

Conversion factors (px_per_mm_x, px_per_mm_y) were calculated from the smallest grid elements and provided within each task prompt.

Amplitude precision was standardized at 0.01 mV; temporal precision at 4 ms (0.1 mm).

S1.5. Waveform Definitions

Wave definitions adhered strictly to conventional electrophysiological standards:

- QRS complex: high-slope deflection; anchor for temporal alignment;
- P-wave search window: 200 ms (5 mm) preceding QRS onset;
- P-wave onset: first deviation from baseline within the search window;
- P-wave offset: return to baseline before the PR segment;

- Baseline: median of a low-slope segment surrounding the P wave;
- P-wave duration: onset-to-offset interval (ms);
- P-wave amplitude: maximum absolute excursion from baseline (mV).

S1.6. Measurement Procedure

For each lead, the model:

1. Detected QRS complexes;
2. Identified P waves for five consecutive sinus beats;
3. Measured duration and amplitude for each beat;
4. Computed mean and standard deviation;
5. Assigned a quality flag if the P wave was not measurable.

If a lead could not be reliably measured, it was excluded from the corresponding derived index.

S1.7. Derived Indices

The following indices were computed:

- mean P-wave amplitude (mean_PwA_mV);
- maximum P-wave amplitude (max_PwA_mV);
- total P-wave dispersion (PwD_disp_total_ms);
- limb-lead dispersion (PwD_disp_limb_ms);
- precordial-lead dispersion (PwD_disp_precordial_ms);
- P-wave Vector Magnitude (PwVM_mV):
  $PwVM = \sqrt{(PwA\_DII^2 + PwA\_V6^2 + (0.5 \times PwA\_V2)^2)}$.

S1.8. JSON Output Requirements

The model returned a structured JSON object containing:

- ECG identifier;
- lead-specific P-wave metrics (mean, SD, quality flag);
- global derived indices.

No free text or commentary was permitted in the model output.

S1.9. JSON Output

Below is the exact JSON template used for AI-based ECG analysis.
All variables, calibration parameters, quality flags, and derived indices follow the mandatory schema defined in the prompting system.


```
{
  "ecg_id": "ECG_001",
  "calibration": {
    "paper_speed_mm_s": 25,
```

"voltage_gain_mm_mV": 10,
"px_per_mm_x": 12.47,
"px_per_mm_y": 12.50,
"amplitude_resolution_mV": 0.01,
"time_resolution_ms": 4
},
"leads": {
"I": {
"PwD_ms_mean": null,
"PwD_ms_sd": null,
"PwA_mV_mean": null,
"PwA_mV_sd": null,
"quality_flag": "not_measurable"
},
"II": {
"PwD_ms_mean": 114,
"PwD_ms_sd": 6,
"PwA_mV_mean": 0.19,
"PwA_mV_sd": 0.02,
"quality_flag": "ok"
},
"III": {
"PwD_ms_mean": 102,
"PwD_ms_sd": 4,
"PwA_mV_mean": 0.11,
"PwA_mV_sd": 0.01,

"quality_flag": "ok"
},
"aVR": {
"PwD_ms_mean": null,
"PwD_ms_sd": null,
"PwA_mV_mean": null,
"PwA_mV_sd": null,
"quality_flag": "not_measurable"
},
"aVL": {
"PwD_ms_mean": 98,
"PwD_ms_sd": 5,
"PwA_mV_mean": 0.09,
"PwA_mV_sd": 0.01,
"quality_flag": "ok"
},
"aVF": {
"PwD_ms_mean": 106,
"PwD_ms_sd": 4,
"PwA_mV_mean": 0.15,
"PwA_mV_sd": 0.01,
"quality_flag": "ok"
},
"V1": {
"PwD_ms_mean": 92,
"PwD_ms_sd": 6,

"PwA_mV_mean": 0.12,
"PwA_mV_sd": 0.02,
"quality_flag": "ok"
},
"V2": {
"PwD_ms_mean": 96,
"PwD_ms_sd": 5,
"PwA_mV_mean": 0.10,
"PwA_mV_sd": 0.01,
"quality_flag": "ok"
},
"V3": {
"PwD_ms_mean": 104,
"PwD_ms_sd": 4,
"PwA_mV_mean": 0.14,
"PwA_mV_sd": 0.01,
"quality_flag": "ok"
},
"V4": {
"PwD_ms_mean": 110,
"PwD_ms_sd": 4,
"PwA_mV_mean": 0.18,
"PwA_mV_sd": 0.02,
"quality_flag": "ok"
},
"V5": {

"PwD_ms_mean": 112,
"PwD_ms_sd": 5,
"PwA_mV_mean": 0.20,
"PwA_mV_sd": 0.02,
"quality_flag": "ok"
},
"V6": {
"PwD_ms_mean": 116,
"PwD_ms_sd": 6,
"PwA_mV_mean": 0.22,
"PwA_mV_sd": 0.02,
"quality_flag": "ok"
}
},
"derived_indices": {
"mean_PwA_mV": 0.16,
"max_PwA_mV": 0.22,
"PwD_disp_total_ms": 24,
"PwD_disp_limb_ms": 16,
"PwD_disp_precordial_ms": 20,
"PwVM_mV": 0.29
}
}

S1.10. Reproducibility

The complete prompting workflow, including system prompts, task prompts, calibration instructions, and schema definitions, is provided to ensure full reproducibility across centers and software versions.
