# Supplementary material for: Artificial Intelligence-Based Evaluation of Post-Procedural Electrocardiographic Parameters to Identify Patients at Risk of Atrial Fibrillation Recurrence After Transcatheter Ablation
Source: J Clin Med. 2025 Nov 20;14(22):8248. doi: 10.3390/jcm14228248 (PMC12653835; doi:10.3390/jcm14228248)
Supplement: Supplementary file 1 [file jcm-14-08248-s001.zip › jcm-3962775-supplementary/Table S1.pdf]

Table s1. Incremental effect of P-wave amplitude in lead II on the odds of atrial fibrillation (AF) recurrence.

Increment (mV)	Exp(B·Δx)	95% CI Lower	95% CI Upper
0.1	1.07e+01	1.48	7.68e+01
0.2	1.14e+02	2.20	5.89e+03
0.3	1.21e+03	3.26	4.52e+05
0.4	1.30e+04	4.84	3.47e+07
0.5	1.38e+05	7.17	2.66e+09

Values represent odds ratios (Exp(B·Δx)) for increasing increments of P-wave amplitude (0.1–0.5 mV), with corresponding 95% confidence intervals. Odds ratios increase exponentially with higher P-wave amplitude, indicating progressively greater risk of AF recurrence at one-year follow-up.
